# Natural Antioxidants: A Review of Studies on Human and Animal Coronavirus

**DOI:** 10.1155/2020/3173281

**Published:** 2020-08-12

**Authors:** Lúcio Ricardo Leite Diniz, Carlos da Silva Maia Bezerra Filho, Burtram C. Fielding, Damião Pergentino de Sousa

**Affiliations:** ^1^College of Nordeste da Bahia, Coronel João Sá, Bahia, Brazil; ^2^Department of Pharmaceutical Sciences, Federal University of Paraíba, João Pessoa, PB, Brazil; ^3^Molecular Biology and Virology Research Laboratory, Department of Medical Biosciences, University of the Western Cape, Cape Town, South Africa

## Abstract

The outbreaks of viruses with wide spread and mortality in the world population have motivated the research for new therapeutic approaches. There are several viruses that cause a biochemical imbalance in the infected cell resulting in oxidative stress. These effects may be associated with the development of pathologies and worsening of symptoms. Therefore, this review is aimed at discussing natural compounds with both antioxidant and antiviral activities, specifically against coronavirus infection, in an attempt to contribute to global researches for discovering effective therapeutic agents in the treatment of coronavirus infection and its severe clinical complications. The contribution of the possible action of these compounds on metabolic modulation associated with antiviral properties, in addition to other mechanisms of action, is presented.

## 1. Introduction

Coronaviruses (CoVs) belong to a family of enveloped viruses with a positive sense, single-stranded RNA genome. CoVs cause illness ranging from upper respiratory tract infections (URTIs) resembling the common cold to lower respiratory tract infections (LRTIs) such as bronchitis, pneumonia, and even severe acute respiratory syndrome (SARS) with most serious disease outcomes in the elderly, immunocompromised patients, and infants [1, 2]. HCoV-OC43 (OC43), HCoV-229E (229E), HCoV-NL63 (NL63), and HCoV-HKU1 (HKU1) were the first documented human CoVs (HCoVs), which usually cause URTIs and less frequently are associated with LTRI diseases [3]. In the last decades, two human coronaviruses created great concern for the world medical community due to significant disease and mortality [4, 5]. In 2003, severe acute respiratory syndrome-coronavirus (SARS-CoV) was characterized by acute atypical pneumonia and diffuse alveolar damage (DAD) in roughly 8000 patients and with almost 800 deaths, representing a nearly 10% mortality rate [6]. More recently, in 2012, a new human coronavirus, designated as Middle East respiratory syndrome-coronavirus (MERS-CoV), was identified, and the global ongoing outbreak of MERS with over 2519 official cases and 866 deaths represented approximately 34% case fatality rate to date in humans [7].

Over the last few months, a new strain of human coronavirus, SARS-CoV-2 (also known as 2019-nCoV), has caught the world's seven continents' attention with its rapid global spread, affecting at least 200 countries and territories, infecting more than 3,000,000 and claiming more than 202,597 lives worldwide [8]. The coronavirus pandemic has promoted isolation and uncertainly fear and panic worldwide. In addition, it will likely lead to changes in political and economic power in ways that can be determined only later [9].

It is important to note that there are many similarities among different coronavirus species, but not in all aspects. Depending on the molecular mechanism of viral inhibition promoted by an antiviral agent, the analysis of the data and comparison between animal and human CoVs must be done very carefully. In fact, it is important to note that there are differences between human and animal CoV receptors, which will likely result in different affinities, or unlikely interactions, of an antiviral agent with the different CoV receptors. However, if the antiviral agent interferes with the replication and/or assembly of the CoVs, there is a higher probability of obtaining similar antiviral activity results in human CoV tests [1, 2, 10, 11]. Following this line, our search in specialized literature was focused, mainly, on studies that investigated the anticoronavirus effects of natural antioxidants by inhibiting proteases for viral replication.

## 2. Materials and Methods

The present study was carried out based on a search of the literature of natural antioxidants and coronavirus. The search, performed in the PubMed database, included studies published until March 2020 and used the following keywords: coronavirus, antioxidants, flavonoids, oxidative stress, MERS-CoV; SARS-CoV, 229E, NL63, OC43, HKU1, MERS-CoV virus infection; and Middle East Respiratory Syndrome Virus. The scientific publications were selected from studies published in the English language.

## 3. Pathogenic Mechanism of Coronavirus-Induced Cell Damage

The high mortality rate associated with the three pathogenic HCoVs has been mainly attributed to the development of digestive and respiratory tract injuries observed following infection. Acute atypical pneumonia and diffuse alveolar damage that progress to deposition of fibrous tissue, denuded airways, haemorrhage, and elevated macrophage infiltration are sometimes accompanied by watery diarrhoea, dehydration, and vomiting [2, 12, 13].

Despite the molecular mechanisms of coronavirus-induced intestine and lung pathogenesis not fully elucidated and still unclear, studies have suggested that late-term disease progression is unrelated to viremia. It is now believed more likely to be associated with the immunopathological mechanism [14, 15]. Viral clearance and subsequent recovery from infection require activation of an effective host immune response; however, many immune effector cells may also cause injury to host tissues [16]. Together with inflammatory and immune response signaling, the presence of oxidative compounds, such as reactive oxygen species (ROS), plays important roles in the pathogenic mechanism of cell damage induced by CoVs through oxidative stress [17].

Oxidative stress is defined as an interruption and/or deregulation of the signaling and redox system that can be caused by an imbalance in the production of oxidant and antioxidant species [18]. Among the main oxidant agents, ROS and reactive nitrogen species (RNS) stand out. In order to counterbalance the oxidant species, there is an antioxidant system formed by enzymes and nonenzymatic molecules [19, 20]. However, during pathological events, such as viral infections, there may be an increase in the production of oxidant species not neutralized by the antioxidant system, resulting in oxidative stress that promotes cellular damage through protein denaturation, changes in the functions of nucleic acids, lipid peroxidation, and cell death [21–23].

In addition, during viral infection, oxidative stress contributes to viral pathogenesis through stimulating inflammation, loss of immune function, and increased viral replication that may occur due to the activation of the nuclear factor kappa B (NF-*κ*B) transcription pathway [24–26]. Current evidence suggests that cytokine dysregulation—also called cytokine storm—contributes to severe disease caused by the pathogenic CoVs [27, 28]. The exact mechanisms are not clear yet, but research on influenza A virus shows that infection causes a rapid influx of inflammatory cells. This is followed by an increase in reactive oxygen species production and cytokine expression and release, which ultimately leads to acute lung injury [29]. In general, RNA viruses promote changes in the body's antioxidant defense system, affecting enzymes such as superoxide dismutase (SOD) and catalase (CAT), in addition to reducing the levels of antioxidant molecules such as ascorbic acid, carotenoids, and reduced glutathione (GSH) [30–32]. Wu et al. reported that glucose-6-phosphate dehydrogenase- (an important antioxidant enzyme that produces NADPH) knockdown cells were more susceptible to infection by HCoV-229E than normal cells [33]. Interestingly, Ye and colleagues have reported that the inhibition of ROS production alleviates inflammation caused by influenza A virus infections [29].

In an experimental model of SARS-induced acute lung injury in mice, it was noted that phospholipid oxidation, due to oxidative stress, is one of the main triggering factors of acute lung injury. This happens through the activation of the innate immune response, culminating in the activation of pulmonary macrophages via TLR4-TRIF-TRAF6-NF-*κ*B signaling [17]. Furthermore, hypoxia caused by acute lung injury can cause myocardial injury due to the production of ROS, aggravating infections caused by coronavirus disease 2019 (COVID-19) [34].

Mitochondria have an essential function in energy generation, and for this reason, their function and integrity are strictly regulated in order to respond to varying energy requirements and environmental conditions [35]. Mitochondria are known to function as the control point in apoptotic pathways, releasing proapoptotic factors, mainly ROS, which function as a signaling molecule that may result in cell death [36, 37]. Some studies have shown a relationship between coronavirus infection and dysfunctional or damaged mitochondria, leading to the release of ROS and other proapoptotic substances [38, 39]. In a recent study, Xu et al. reported that ROS and p53 play key roles in regulating many kinds of the cell process during coronavirus infection in Vero cells. According to the authors, coronavirus infection appears to induce a time-dependent ROS accumulation, which in turn is linked to regulatory mechanisms of p53 activation and apoptosis in infected cells [40].

Antioxidant substances promote improvement in cases of disease caused by coronaviruses, such as apolipoprotein D—a lipocalin that promoted a neuroprotective effect against encephalitis induced by human coronavirus OC43—in mice. This protective effect occurred through the reduction of oxidative stress, cerebral lipid peroxidation, and regulation of inflammation [41, 42]. Also, the treatment with antioxidants, such as pyrrolidine dithiocarbamate or N-acetylcysteine, significantly inhibits coronavirus-induced apoptosis [43]. Moreover, melatonin promotes downregulation of acute lung oxidative injury due to its anti-inflammatory and antioxidant actions, making it a possible compound in the treatment of COVID-19 [44]. Based on these studies, compounds that have antioxidant actions can be helpful in the treatment of infections promoted by coronavirus.

**Figure 1:**
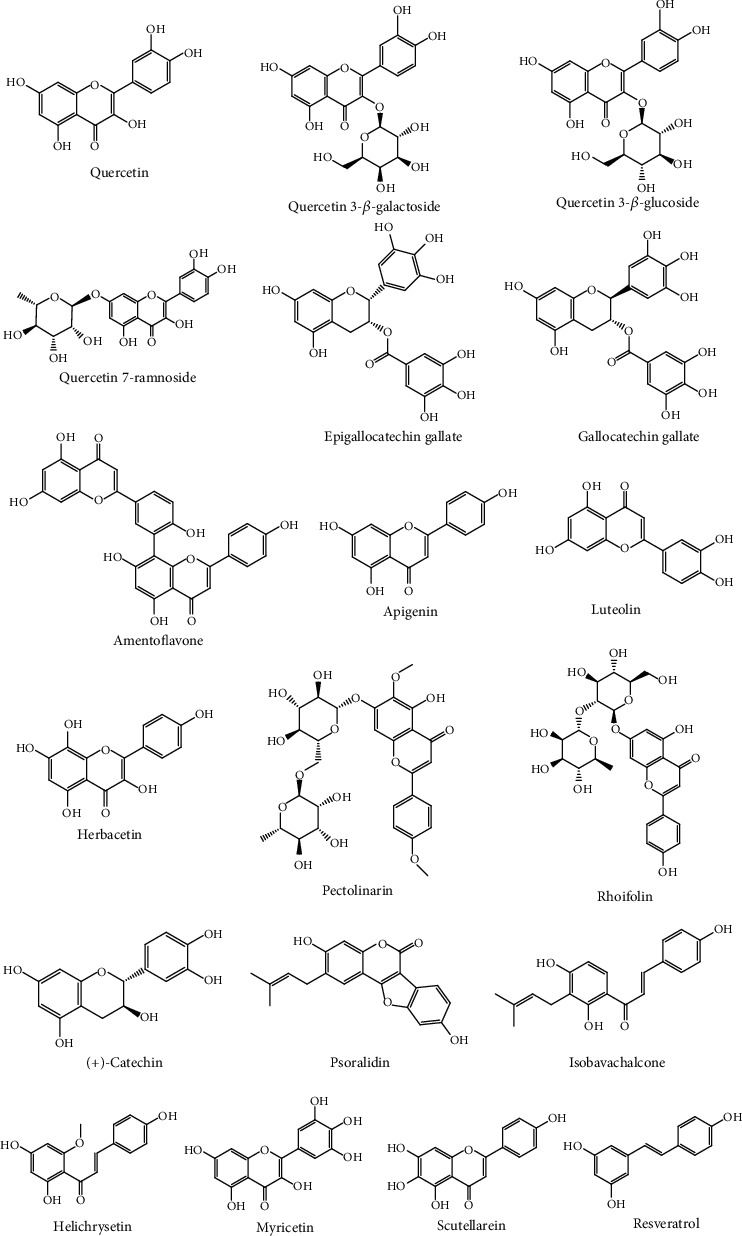
Chemical structures of bioactive antioxidants against coronavirus.

In general, antioxidant properties of polyphenolic compounds, such as some flavonoids, have been associated with the presence of aromatic phenolic rings that promote the electron donation and hydrogen atom transfer to free radicals, acting as free radical scavengers, reducing agents, and quenchers of single oxygen formation [45]. Thus, the aim of this study was to investigate the antioxidant capacity and antiviral activity of natural antioxidants against coronavirus. The compounds are illustrated in Figure 1.

## 4. Occurrence and Antioxidant Properties of Anticoronavirus Compounds

Quercetin can be found in plants such as *Rubus fruticosus* L. and *Lagerstroemia speciosa* (L.) Pers. [46, 47]. Also, quercetin shows antioxidant activity at a concentration of 10 *μ*mol/L in HepG2 cells, inhibiting oxidative stress promoted by H_2_O_2_ [48], promotes an increase in SOD, CAT, and glutathione peroxidase (GPx), and reduces lipid peroxidation in rats with chronic prostatitis/chronic pelvic pain syndrome [49]. Moreover, quercetin improves sepsis-induced acute lung injury in rats, by reducing lipid peroxidation and inflammation and increasing SOD and CAT levels [50].

In addition, quercetin glycosides with antioxidant activity, such as quercetin 3-*β*-glucoside, have already been isolated from plants such as *Passiflora subpeltata* Ortega and *Chamomilla suaveolens* (Pursh) Rydb. [51, 52]. The administration of quercetin 3-*β*-glucoside (40 mg/kg p.o.) in streptozotocin-induced diabetic rats promotes an increase in the levels of antioxidant enzymes (SOD, CAT, and GPx) and nonenzymatic antioxidants (vitamins C and E and GSH) and a reduction of lipid peroxidation [53]. Quercetin 3-*β*-galactoside (hyperoside) is found mainly in plants of the *Hypericum* genus such as *Hypericum perforatum* L. [54, 55]. Moreover, it showed cardioprotective activity in high glucose-induced injury of myocardial cells through decreased apoptosis and ROS production and increased SOD levels [56]. Quercetin 7-ramnoside is also found in plants of the *Hypericum* genus such as *Hypericum japonicum* Thunb. ex Murray. This flavonoid shows hepatoprotective activity against carbon tetrachloride in mice by decreasing lipid peroxidation and increasing CAT and GSH levels, in addition to presenting values of 118.75 *μ*M and 128.47 *μ*M in the 2,2-diphenyl-1-picrylhydrazyl (DPPH) and 2,2′-azino-bis-3-ethylbenzthiazoline-6-sulphonic acid (ABTS) assays, respectively [57].

Epigallocatechin gallate is present in *Parkia roxburghii* G. Don and is one of the main metabolites found in green tea and Liubao tea (*Camellia sinensis* LO Kuntze). Also, gallocatechin gallate can be found in this plant [58–61]. Literature data reveal that the administration (i.p.) of 2.5 mg/100 g of epigallocatechin gallate in rats, with streptozotocin-induced diabetes mellitus, promotes a reduction in oxidative stress through reductions in parameters such as indirect nitric oxide synthesis and status total oxidative, as well as an increase in levels of CAT and total antioxidant capacity of plasma [62]. Furthermore, it promotes cardioprotection by antioxidant mechanisms [63].

Green tea has a high antioxidant capacity, due to the high levels of catechins present [64]. He and collaborators compared the antioxidant activities of catechins and reported that epigallocatechin gallate has greater antioxidant activity via radical scavenging activity (400 *μ*M) with values of 77.2 ± 4.3%, 90.2 ± 3.1%, and 100 ± 3.1% compared to its epimer, gallocatechin gallate, with values of 68.2 ± 3.4%, 82.2 ± 3.8%, and 95.5 ± 3.9% in the DPPH, ABTS, and ferric reducing antioxidant power (FRAP), respectively [65].

Amentoflavone is a biflavonoid present in leaves of *Ginkgo biloba* L., *Garcinia brasiliensis* L., and *Nandina domestica* L. [66–68]. This biflavonoid has a high antioxidant capacity (19.21–75.52%), demonstrated in scavenging tests of DPPH, ABTS, superoxide, and hydroxyl radicals [67]. Moreover, amentoflavone prevents acute lung injury induced by sepsis in rats by decreasing thiobarbituric acid reactive substance (TBARS) levels and by increasing levels of SOD and GSH [69].

Apigenin is mainly present in flowers and leaves, being abundantly found in *Apium graveolens* L., *Petroselinum crispum* (Mill.) Fuss, and *Matricaria chamomilla* L. [70]. Sánchez-Marzo and collaborators evaluated the antioxidant capacity of apigenin using the Trolox equivalent antioxidant capacity (TEAC), oxygen radical absorbance capacity (ORAC), and FRAP assays. The results show that apigenin has good antioxidant activity with values of 2022.2 ± 154.8 *μ*mol TE^a^/mmol, 887.9 ± 5.8 *μ*mol TE^a^/mmol, and 113.2 ± 12.2 *μ*mol Fe^2+^/mmol, respectively [71]. In addition, oral administration of apigenin 25 mg/kg/day for 12 days in an experimental model of cardiotoxicity induced by doxorubicin in rats promoted cardioprotection by reducing levels of malondialdehyde (MDA), increasing SOD levels, and preventing cardiomyocyte apoptosis [72].

Luteolin is present in foods such as carrot, cabbage, tea, and apple and is found in *Ugni molinae* Turcz. [73, 74]. Data show that luteolin (50 *μ*g/mL) increases the levels of GSH, the expression of GSH synthetase, and the activity of SOD and CAT in human colon cancer cells (HT-29) [75]. Furthermore, luteolin attenuates the sepsis-induced acute lung injury in mice by reducing lipid peroxidation and increasing SOD and CAT activity, in addition to suppressing the NF-*κ*B pathway [76].

Herbacetin is ubiquitous in plants of the genus *Rhodiola*, such as *Rhodiola rosea* L. [77]. Herbacetin glycosides are also present in the roots of *R. sachalinensis* A. Bor and show antioxidant activity [78]. Veeramani et al. reported that the administration of herbacetin (40 mg/kg p.o.) in mice, with obesity-associated insulin resistance, promotes an increase in the activity of the enzyme glucose-6-phosphate dehydrogenase, which is directly related to the production of NADPH [79].

Pectolinarin is present in plants of the genus *Cirsium* such as *Cirsium setidens* Nakai and *Cirsium japonicum* DC. The administration of pectolinarin (10 and 20 mg/kg, p.o. for two weeks) in rats promotes antioxidant effects in hepatic injury induced by D-galactosamine by increasing levels of SOD, GSH, glutathione reductase, and glutathione S-transferase [80, 81].

Rhoifolin is found in citrus fruits, such as *Citrus limetta* Risso. Studies have indicated that its radical peroxyl scavenging capacity is higher than Trolox in ORAC assays (approximately 10 Trolox equivalents (*μ*M)) [82, 83].

Meanwhile, the (+)-catechin is a flavonoid present in leaves of green tea, wine, and fruits [84, 85]. Grzesik et al. investigated the antioxidant action of catechin through the ABTS scavenging activity and FRAP tests. The results show values of 3.965 ± 0.067 (mol Trolox equivalents/mol) and 0.793 ± 0.004 (mol Trolox equivalents/mol), respectively. In addition, catechin shows greater protective properties in the dihydrorhodamine 123 oxidation assay (IC_50_0.805 ± 0.072 *μ*M) than GSH and ascorbic acid (14.1 and 13.9 *μ*M, respectively) [86].

Psoralidin is a prenylated coumestan, which is found in plants of the Fabaceae family, such as *Psoralea corylifolia* L. Xiao and collaborators, investigating the antioxidant potential of compounds isolated from *P. corylifolia*, observed that psoralidin shows the best antioxidant activity by the method of electron spin resonance spectroscopy with an IC_50_ value of 44.7 *μ*M [87].

The compound isobavachalcone has been isolated from plants of the Fabaceae and Moraceae families [88, 89]. Isobavachalcone shows a strong antioxidant activity in DPPH SC_50_, FRAP, and ABTS SC_50_ assays with values of 250.8 *μ*M, 0.4 ± 0.05 mM equivalent to FeSO_4_·7H_2_O, and 510.1 mM, respectively. In addition, the compound has been reported to inhibit the NF-*κ*B pathway in Sephadex-induced lung injury in rats [88, 90].

Helichrysetin is a chalcone that is found in plants of the *Helichrysum* genus such as *Helichrysum odoratissimum* L. [91]. In a study investigating the antioxidant activity of natural and prenylated chalcones, Vogel et al. found that helichrysetin is the substance that shows the highest antioxidant activity in the ORAC test with values of 4.4 ± 0.6 Trolox equivalents [92].

Myricetin is widely found in the plant families Myricaceae and Anacardiaceae and is widely used as health food supplement due to its antioxidant properties [93, 94]. Bennett et al. demonstrated that myricetin reacts with oxygen-centered galvinoxyl radicals more than 28 times higher than vitamin E (*d*-alpha-tocopherol). Furthermore, myricetin was able to scavenge 21% and 54% on the DPPH assay (5 *μ*g/mL and 10 *μ*g/mL, respectively) [95]. Interestingly, the compound prevents DNA damage, by lipid peroxidation and increasing the activity of SOD, CAT, and GPx in Chinese hamster lung fibroblast cells (V79-4) treated with H_2_O_2_ [96].

Scutellarein is found in *Scutellaria barbata* D. Don and *Polygonum viscosum* Buch-ham [97, 98]. Liu et al. investigated the antioxidant activity of scutellarein through the DPPH, ABTS, and superoxide scavenging assays. They noted that the compound shows good antioxidant activity with values of 18.7 ± 0.1 *μ*M, 18.3 ± 1.2 *μ*M, and 79.0 ± 0.5 *μ*M, respectively, while the Trolox, a standard antioxidant compound, presented 20.2 ± 0.5 *μ*M, 23.7 ± 0.4 *μ*M, and 291.5 ± 40.6 *μ*M, respectively [99].

**Figure 2:**
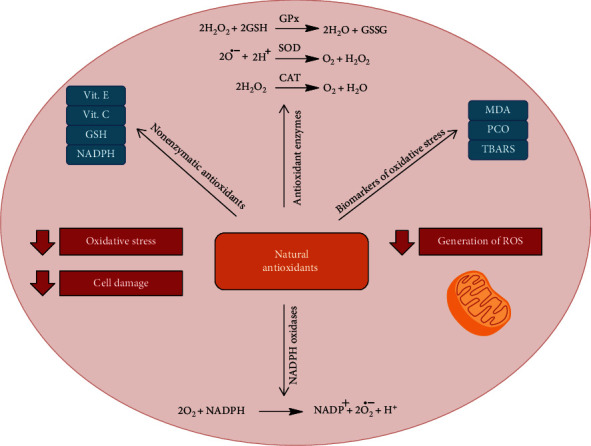
The main antioxidant mechanisms of natural compounds reported in this review. Dashed line: inhibition. Full line: activation.

Resveratrol is found in grapes, peanuts, and blueberries and can be isolated from *Veratrum grandiflorum* O. Loes [100]. Literature shows that resveratrol has good antioxidant activity with DPPH SC_50_/*r*^2^ values of 26.37/0.849 *μ*mol/dm. Moreover, it is able to reduce the production of ROS by inhibiting the activity and expression of NADPH oxidases, by eliminating oxidant agents, including radical hydroxyl, superoxide, hydrogen peroxide, and peroxynitrite [101, 102]. The treatment of resveratrol (50 mg/kg p.o.) in rats reduces oxidative stress in obstructive lung disease by increasing SOD activity and reducing MDA levels, indicating a decrease in lipid peroxidation [103].  Table 1 shows the main actions of natural antioxidants discussed in this study, and Figure 2 illustrates these activities.

## 5. Effect of Natural Antioxidants in Coronavirus Infections

This review focused on studies reporting on the anticoronavirus activity of natural antioxidants. Based on exclusion criteria, data from nineteen compounds were discussed.

The oxidative stress pathway could potentially be a key element in coronavirus-induced apoptosis and pathogenesis [104]. For this reason, it is interesting to investigate the use of antioxidants as potential therapeutic tools—either as an alternative or as an adjuvant to conventional therapies—in the treatment of coronavirus infections. Among the antioxidant compounds evaluated as for coronavirus infections are the flavonoids, which are compounds widely found in fruits, vegetables, and certain beverages. In fact, research groups have reported that antioxidant flavonoids, including (+)-catechin, luteolin, apigenin, quercetin, and quercetin 7-rhamnoside, inhibit ROS accumulation and apoptosis of cells infected with different coronavirus, including porcine epidemic diarrhoea coronavirus (PEDV) and transmissible gastroenteritis coronavirus (TGEV) [105–107].

As shown with the recent COVID-19 pandemic, the search for alternative or new antiviral therapies for the treatment of coronavirus diseases remains important. Based on the literature, antioxidant therapies offer an attractive option.

The high number of deaths and clinical complications observed in SARS- and MERS-CoV epidemics motivated the search for effective therapeutic agents. This was necessitated when many of the tested conventional drugs and antiviral therapies proved ineffective in treating SARS-CoV infections. For example, the initial treatment of SARS-CoV with antiviral agents such as ribavirin and corticosteroids did not achieve very satisfactory results, mainly because corticosteroids exert immunosuppressor effects on the humoral and cellular immune systems [108, 109]. Other drugs such as pentoxifylline were considered for the treatment of SARS due to its interesting therapeutic properties that include anti-inflammatory, antiviral, immunomodulatory, and bronchodilatory effects. However, it too was not successful in the clinical treatment of SARS-CoV infection [110].

Many antioxidant compounds show antiviral activity against SARS-CoV. The antiviral activity has been mainly attributed to the inhibition of the 3C-like protease (3CL^pro^) of SARS-CoV, a vital enzyme for SARS-CoV replication [111]. As an example, multiples studies have reported that quercetin and quercetin-derived compounds, such as quercetin 3-*β*-galactoside, display potent 3CL^pro^ inhibitory e5ffect and consequent reduction of SARS-CoV replication [112]. Other antioxidants, such as epigallocatechin gallate, gallocatechin gallate, amentoflavone, apigenin, luteolin, herbacetin, rhoifolin, and pectolinarin, are also found to efficiently block the enzymatic activity of SARS-CoV 3CL^pro^ [111, 113, 114].

Moreover, some natural antioxidants exhibit promising antiviral activity against SARS-CoV infection by interfering with different targets involved in SARS-CoV replication, in particular the SARS-CoV papain-like protease (PL^pro^) and SARS-CoV helicase protein. Kim et al. reported that isobavachalcone and psoralidin inhibit PL^pro^ in a dose-dependent manner with IC_50_ ranging between 4.2 and 38.4 *μ*M [115]. Previously, Yu et al. reported that myricetin and scutellarein potently inhibit the SARS-CoV helicase protein *in vitro* by affecting the ATPase activity [116].

MERS-CoV is another zoonotic coronavirus transmitted between animals and human beings that causes severe morbidity and mortality. No antiviral medicines with satisfactory efficacy for the treatment of MERS-CoV-infected patients have been identified to date. Similar to SARS-CoV, natural antioxidant libraries have been probed for potential inhibitory compounds against MERS-CoV 3C-like protease. Jo et al. showed that herbacetin, isobavachalcone, quercetin 3-*β*-d-glucoside, and helichrysetin, four compounds with recognized antioxidant activity, can block the enzymatic activity of MERS-CoV 3CL^pro^ using a tryptophan-based fluorescence method. Furthermore, the experimental and computational studies show that flavonol and chalcone are favourite scaffolds to bind with the catalytic site of MERS-CoV 3CL^pro^ [117].

**Figure 3:**
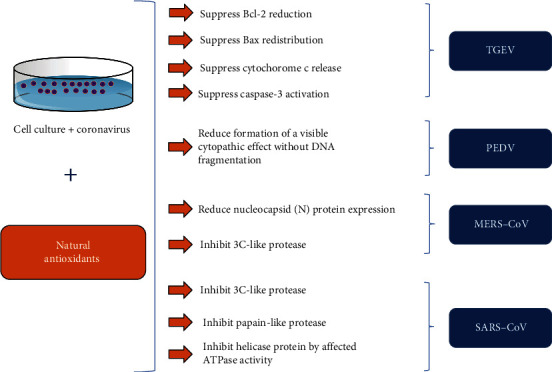
Inhibitory actions of natural antioxidants against coronavirus.

In a study performed by Lin et al., the antiviral activities of resveratrol were investigated in MERS-infected Vero E6 cells. The authors reported a significant inhibition of MERS-CoV infection and prolonged host cell survival after virus infection, which they speculate was promoted by resveratrol. In addition, they also found that the expression of the nucleocapsid (N) protein, which is essential for MERS-CoV replication, is decreased after resveratrol treatment [118]. It is important to mention that *in vitro* models of coronavirus infection also show antiviral activity of flavonoids extracted from flowering cherry cultivars and black tea [119, 120]. Finally, antioxidants, such as resveratrol, also are able to block infection produced by herpesvirus [121, 122]. The discovery of antiviral compounds from a bioactive compound against other viruses is an interesting strategy for obtaining new antiviral drugs.  Table 2 shows the main actions of the natural antioxidants against the coronavirus, and Figure 3 summarizes these activities.

## 6. Conclusions

In conclusion, this review shows that antioxidant compounds, prominently flavonoids, exhibit antiviral action in models of coronavirus infections. In general, the antiviral activity might be attributed, at least in part, to the inhibitory effect on the enzymatic activity of targets involved in coronavirus replication, including SARS-CoV 3CL^pro^, SARS-CoV papain-like protease (PL^pro^), SARS-CoV helicase protein, and MERS-CoV 3CL^pro^. In addition, some studies provide evidence that the reduction of ROS accumulation retards the coronavirus-activated apoptotic signaling. Therefore, the mechanisms of oxidative stress could be the key element to be studied in coronavirus infections, including those related to inflammatory processes arising from the action of this virus. Obviously, further investigations are needed to elucidate other pharmacological mechanisms by which natural antioxidants play an antiviral effect. Despite the findings reported in this review, they cannot be generalized to COVID-19. However, the data provided support to the investigation of natural antioxidants as a potential therapeutic approach in the treatment for COVID-19 and its severe clinical complications, either as an alternative or as an adjuvant to conventional therapies, and contribute to the search for new prototypes in the development of drugs against coronavirus infections.

## Figures and Tables

**Table 1:** Antioxidant properties of natural inhibitors of coronavirus.

Compound	Type of cells tested/assays/experimental models	Concentration / dose	Antioxidant effect	Reference
Quercetin	HepG2 cells	10 *μ*mol/L	Inhibiting oxidative stress promoted by H_2_O_2_	[48]
	Rats with chronic prostatitis/chronic pelvic pain syndrome	50 mg/kg (p.o.)	Promoted an increase in SOD, CAT, and GPx and reduced lipid peroxidation	[49]
	Sepsis-induced acute lung injury in rats	100 mg/kg (p.o.)	Reduces lipid peroxidation and increases SOD and CAT levels	[50]
Quercetin 3-*β*-glucoside	Streptozotocin-induced diabetic rats	40 mg/kg (p.o.)	Increases levels of SOD, CAT, GPx, vitamins C and E, and GSH and reduces lipid peroxidation	[53]
Quercetin 3-*β*-galactoside	High glucose-induced injury of myocardial cells	20 nmol/L	Decreases apoptosis and ROS production and increases SOD levels	[56]
Quercetin 7-ramnoside	CCl_4_-induced liver damage model in mice DPPH ABTS	20 mg/kg IC_50_ = 118.75 *μ*M (DPPH) EC_50_ = 128.47 *μ*M (ABTS)	Decreases lipid peroxidation and increases CAT and GSH levels Scavenging of free radicals	[57]
Epigallocatechin gallate	Rats with streptozotocin-induced diabetes mellitus	2.5 mg/100 g (i.p.)	Reduces indirect nitric oxide synthesis and total oxidative status Increased levels of CAT and total antioxidant capacity of plasma	[62]
	Rats with streptozotocin-nicotinamide-induced diabetes mellitus	2 mg/kg (p.o.)	Increased levels of CAT, SOD, and GSH Reduced levels of superoxide and protein carbonyl (PCO) and prevented DNA damage	[63]
Epigallocatechin gallate	DPPH ABTS FRAP	400 *μ*M	77.2 ± 4.3%, 90.2 ± 3.1%, and 100 ± 3.1%, respectively 68.2 ± 3.4%, 82.2 ± 3.8%, and 95.5 ± 3.9%, respectively	[65]
Gallocatechin gallate	Acute lung injury induced by sepsis in rats	50 mg/kg	Decreases TBARS levels and increases levels of SOD and GSH	[69]
Amentoflavone	DPPH, ABTS, superoxide, and hydroxyl radicals	50 *μ*g/mL	Scavenging of free radicals (19.21-75.52%)	[67]
Apigenin	TEAC ORAC FRAP	2022.2 ± 154.8 *μ*mol TE^a^/mmol, 887.9 ± 5.8 *μ*mol TE^a^/mmol, and 113.2 ± 12.2 *μ*mol Fe^2+^/mmol, respectively	Scavenging of free radicals	[71]
	Cardiotoxicity induced by doxorubicin in rats	25 mg/kg (p.o.)	Reduces levels of MDA, increases SOD levels, and prevents cardiomyocyte apoptosis	[72]
Luteolin	Human colon cancer cells (HT-29)	50 *μ*g/mL	Increases levels of GSH, expression of GSH synthetase, and the activity of SOD and CAT	[75]
	Acute lung injury induced by sepsis in mice	0.2 mg/kg (i.p.)	Reduces lipid peroxidation, increases the activity of SOD and CAT, and suppresses the NF-*κ*B pathway	[76]
Herbacetin	Mice with obesity-associated insulin resistance induction	40 mg/kg (p.o.)	Increases the activity of glucose-6-phosphate dehydrogenase	[79]
Pectolinarin	Hepatic injury induced by D-galactosamine in rats	10 and 20 mg/kg (p.o.)	Increases levels of SOD, GSH, glutathione reductase, and glutathione S-transferase	[80]
Rhoifolin	ORAC	Approximately 10 Trolox equivalents (*μ*M)	Scavenging of free radicals	[83]
Catechin	ABTS FRAP Dihydrorhodamine 123 oxidation assay	3.965 ± 0.067 (mol Trolox equivalents/mol), 0.793 ± 0.004 (mol Trolox equivalents/mol), and IC_50_ 0.805 ± 0.072 *μ*M, respectively	Scavenging of free radicals	[86]
Isobavachalcone	DPPH SC_50_ FRAP ABTS SC_50_	250.8 *μ*M, 0.4 ± 0.05 mM equivalent to FeSO_4_·7H_2_O, and 510.1 mM, respectively	Scavenging of free radicals	[88]
Psoralidin	Electron spin resonance	IC_50_ = 4.7 *μ*M	Scavenging of free radicals	[87]
Myricetin	DPPH Chinese hamster lung fibroblast cells (V79-4) treated with H_2_O_2_	5 *μ*g/mL and 10 *μ*g/mL 10 *μ*g/mL	Scavenging of free radicals (21% and 54%, respectively) Prevents DNA damage and lipid peroxidation Increases the activity of SOD, CAT, and GPx	[96]
Helichrysetin	ORAC	4.4 ± 0.6 Trolox equivalents	Scavenging of free radicals	[92]
Scutellarein	DPPH ABTS Superoxide radicals	18.7 ± 0.1 *μ*M, 18.3 ± 1.2 *μ*M, and 79.0 ± 0.5 *μ*M, respectively	Scavenging of free radicals	[99]
Resveratrol	DPPH SC_50_/*r*^2^	26.37/0.849 *μ*mol/dm	Scavenging of free radicals	[101]
	Rats with obstructive lung disease	50 mg/kg	Increases SOD activity and reduces MDA levels	[103]
	Hypercholesterolemic ApoE-KO mouse	100 mg/kg	Inhibits the activity and expression of NADPH oxidases Increases SOD, GPx, and CAT levels	[102]

**Table 2:** Natural antioxidants tested in *in vitro* coronavirus infection models and their main results and mechanism of action.

Antioxidant	Type of cells tested	Concentration (IC_50_)	Antiviral effect	Mechanism of action	Reference
(+)-Catechin	TGEV-infected ST cells	(+)-Catechin (20–80 *μ*M)	Inhibition of TGEV-induced apoptosis	Suppression of the TGEV-induced Bcl-2 reduction, Bax redistribution, cytochrome c release, and caspase-3 activation	[107]
Resveratrol	MERS-infected Vero E6 cells.	Resveratrol (125-250 *μ*M)	Inhibition of MERS-induced infection/apoptosis and prolonged cellular survival after virus infection	Reduction of the expression of nucleocapsid (N) protein essential for MERS-CoV replication	[118]
Quercetin Epigallocatechin gallate Gallocatechin gallate (GCG)	Recombinant 3CL^pro^ was expressed in *Pichia pastoris* GS115	Quercetin (73 *μ*M) Epigallocatechin gallate (73 *μ*M) Gallocatechin gallate (47 *μ*M)	Inhibition of coronavirus replication	GCG displayed a binding energy of -14 kcal mol^−1^ to the active site of 3CL^pro^ and the galloyl moiety at 3-OH position was required for 3CL^pro^ inhibition activity	[114]
Quercetin 7-rhamnoside (Q7R)	PEDV-infected Vero cells	Q7R (10 *μ*M)	Reduction of the formation of a visible cytopathic effect (CPE) without DNA fragmentation	Not specificity	[105, 106]
Amentoflavone Apigenin Luteolin Quercetin Quercetin 3-*β*-galactoside Herbacetin Rhoifolin Pectolinarin	SARS-CoV 3CL^pro^ inhibition using fluorescence resonance energy transfer analysis Molecular docking, SPR/FRET-based bioassays, and mutagenesis Tryptophan-based fluorescence method	Amentoflavone (8.3 *μ*M) Apigenin (208.8 *μ*M) Luteolin (20.2 *μ*M) Quercetin (23.8 *μ*M) Quercetin 3-*β*-galactoside (5-200 *μ*M) Herbacetin (33.17 *μ*M) Rhoifolin (27.45 *μ*M) Pectolinarin (37.78 *μ*M)	Inhibition of SARS-CoV replication	Flavonoids exhibited SARS-CoV 3CL^pro^ inhibitory activity	[113, 112, 111]
Herbacetin, isobavachalcone, quercetin 3-*β*-d-glucoside, helichrysetin	Tryptophan-based fluorescence method	Herbacetin (40.59 *μ*M) Isobavachalcone (35.85 *μ*M) Quercetin 3-*β*-d-glucoside (37.03 *μ*M) Helichrysetin (67.04 *μ*M)	Inhibition of MERS-CoV replication	Flavonoids exhibited MERS-CoV 3CL^pro^ inhibitory activity	[117]
Isobavachalcone Psoralidin	Lineweaver–Burk and Dixon plots	Isobavachalcone (7.30 *μ*M) Psoralidin (4.02 *μ*M)	Inhibition of SARS-CoV replication	Isobavachalcone and psoralidin exhibited SARS-CoV papain-like protease inhibitory activity	[115]
Myricetin, scutellarein	SPR/FRET-based bioassays	Myricetin (2.71 *μ*M) Scutellarein (0.86 *μ*M)	Inhibition of SARS-CoV replication	Myricetin and scutellarein potently inhibit the SARS-CoV helicase protein *in vitro* by affecting the ATPase activity	[116]

## Abbreviations

229E: Human coronavirus-229E
3CL^pro^: 3C-like protease
ABTS: 2,2′-Azino-bis-3-ethylbenzthiazoline-6-sulphonic acid
CoVs: Coronaviruses
COVID-19: Coronavirus disease 2019
CAT: Catalase
DAD: Diffuse alveolar damage
DPPH: 2,2-Diphenyl-1-picrylhydrazyl
FRAP: Ferric reducing antioxidant power
GSH: Reduced glutathione
GPx: Glutathione peroxidase
HCoVs: Human coronaviruses
HKU1: Human coronavirus-HKU1
LRTIs: Lower respiratory tract infections
MDA: Malondialdehyde
MERS-CoV: Middle East respiratory syndrome-coronavirus
NF-*κ*B: Nuclear factor kappa B
NL63: Human coronavirus-NL63
OC43: Human coronavirus-OC43
ORAC: Oxygen radical absorbance capacity
PCO: Protein carbonyl
PEDV: Porcine epidemic diarrhoea coronavirus
PL^pro^: Papain-like protease
RNS: Reactive nitrogen species
ROS: Reactive oxygenated species
SARS: Severe acute respiratory syndrome
SARS-CoV: Severe acute respiratory syndrome-coronavirus
SOD: Superoxide dismutase
TBARS: Thiobarbituric acid reactive substances
TEAC: Trolox equivalent antioxidant capacity
TGEV: Transmissible gastroenteritis coronavirus
URTIs: Upper respiratory tract infections.
